# Validity and Psychometric Properties of the ILO-WHO Workplace Stress Scale: A Study with Workers from the Canary Islands

**DOI:** 10.3390/ejihpe12070051

**Published:** 2022-06-23

**Authors:** Juan Martinez Torvisco, Giuseppe Santisi, Alice Garofalo, Tiziana Ramaci, Massimiliano Barattucci

**Affiliations:** 1 Psicología Cognitiva, Social y Organizacional, Universidad de La Laguna, 38200 San Cristóbal de La Laguna, Spain; jtorvisc@ull.edu.es; 2 Department of Educational Sciences, University of Catania, 95124 Catania, Italy; gsantisi@unict.it; 3 Faculty of Human and Social Sciences, University of Enna “Kore”, 94100 Enna, Italy; alice.garofalo@unikorestudent.it; 4 Department of Human and Social Sciences, University of Bergamo, 24129 Bergamo, Italy; massimiliano.barattucci@unibg.it

**Keywords:** workplace stress, organizational stress, work-related stress, work stress measurement

## Abstract

Occupational stress, as a negative facet, is a pervasive problem with significant implications for organizations, employees, welfare systems and health. The implementation of measurement tools that can capture the different organizational dimensions that determine stress in workers is part of the stress management and troubleshooting strategy that every company must manage daily. The aim of the present study was to adapt and validate the 25-item version of the ILO-WHO stress scale by Ivancevich and Matteson in the context of the Canary Islands of Spain. The tool assesses specific organizational dimensions of work-related stress determinants: organizational climate and structure, leader influence, cohesion, territory, technology and group support. An exploratory factor analysis (EFA) on a sample of 1510 Canary Islands workers was carried out. The results indicate that the job stress scale revealed adequate psychometric properties, construct validity and internal consistency (Cronbach’s alpha = 0.972), and it can be profitably used to measure stress. At the end of the paper, theoretical and practical implications are discussed.

## 1. Introduction

Job stress has received significant and increasing attention over the past 30 years and is now considered a pervasive problem with such significant implications for organizations, employees, welfare systems and health [1] that it has recently been cited as one of the most challenging issues in occupational safety and health [2]. At least in part, this is the result of changing sociopolitical and economic conditions, characterized by discontinuity, flexibility, and continuous adaptation [3,4].

In this scenario, the implementation of measurement tools that can capture the different organizational dimensions (personal, relational, environmental, etc.) determining stress in workers, is now part of the general stress management and troubleshooting strategy that every company must manage daily with the aim of continuous improvement of both work processes and internal relations [5]. The scientific evidence relating to the relationship between work stress and lowering of performance, between occupational stress and physical injuries, cardiovascular disorders, and mental disorders, and between work stress and the worsening of other work outcomes (e.g., satisfaction, commitment, trust, turnover, etc.) is now familiar to most people and to the corporate world in particular [6].

It is also true that the term “job stress” has come to refer to a very varied series of phenomena, constructs, variables, and environmental circumstances at work, ranging from simple perceptions of working conditions to clinical dimensions, real pathologies, and psychological predispositions to events (e.g., coping abilities, problem solving, resilience, self-efficacy, etc.) [7].

Stress at work is universally recognized as the emotional state deriving from perceptions regarding the interaction between an employee and their work environment, which may form a sense of imbalance between perceived resources and organizational demands [8,9]. The effects of stress on workers can be physical (e.g., palpitations), psychological (e.g., anxiety, avoidance) or behavioral (e.g., high turnover, low work performance, arguing with colleagues) [10], and may result in injuries, errors, and conflicts [11].

A review of the literature shows that workplace stress determinants have been investigated primarily in terms of job design and work environment conditions, and different models have been proposed, among which it is worth noting the job demand–control–support model [12,13], which explains mental strain as the result of the interaction between job demand, job control, and job support [14]; and the effort–reward imbalance model [15]. Ivancevich and colleagues [16] proposed two main categories of workplace stressors: the so-called extra-organizational stressors and the worksite stressor, which includes individual, organizational and individual–organization interface stressors [17,18].

Beyond the theoretical framework and the focus on specific determinants, both institutional and scientific indicators clearly underline that, today, the measurement of psychosocial and environmental perceptions related to employee stress is crucial. This is true for monitoring stress, for putting in place change and development policies, and for developing forms of stress management and intervention both at the individual level (development of individual and interpersonal resources that are able to support workers in coping with stress situations and difficulties at work) [19], and at an organizational level (group interventions, work organizational change management, etc.). The practice of monitoring stress represents the efforts of institutions and organizations to ensure healthy working standards, procedures, and environments, and also demonstrates their need to focus on every employee’s health [10].

To date, in the Canary Islands, there has been no validated instrument that would allow the monitoring of a large number of psychosocial variables referring to work conditions and their potential health effects; therefore, the present research was designed to validate the ILO-WHO stress scale in a large sample from this territory.

The Canary archipelago, due to its remote geographical position and its scarcity of natural resources, has an economy more than three-quarters-dependent on the tourism sector; industry is not very extensive and agriculture does not thrive because many products have Spain as their only regular market. The pandemic situation from the end of 2019 resulted in a collapse of the tourism sector which had a negative impact on the economy and on employment. The context-specific elements of occupational stress must therefore be considered in light of the current economic situation in the archipelago.

## 2. How to Measure Stress

The literature on workplace stress is distinguished by being based on several approaches which focus on specific environmental, organizational and relational factors. Some approaches involve the development of direct measures that link stress experienced in the workplace with job design conditions [20]; others involve the implementation of subjective measures which contain constructs related to a theoretical framework (Job Demand-Resources model, effort–reward imbalance model, etc.) [21,22]; still other approaches involve the development of general measures of workplace stress that do not necessarily link to some specific source of stress or to organizational determinants, but instead focus principally on measuring the manifestations of stress (e.g., burnout, physiological responses, workaholism, conflicts, mobbing, etc.) [23,24]. This has produced a real proliferation of workplace stress measures, ranging from very simple measures of workplace stress to complex scales containing many subscales and many organizational determinants of stress (e.g., workload, demands, environmental and working conditions, role/task/job characteristics, managerial support, shifts, etc.) [25,26,27], along with institutional evidence [28], which references 37 instruments used in the measurement of psychosocial hazards in the workplace.

Among the best-known questionnaires are: the Occupational Stress Indicator (OSI) [29], a self-reporting questionnaire which evaluates different aspects of occupational stress (potential sources of job stress, locus of control, job satisfaction and health dimensions); the HSE Management Standards Indicator Tool [30], the Occupational Stress Questionnaire (OSQ) [31], the Job Stress Survey (JSS) [32]; the Job Content Questionnaire (JCQ) [33] which measures job demand and control, and job strain; the Effort–Reward Imbalance (ERI) questionnaire [34], the NIOSH work-related stress questionnaire [35], and many others.

Using the joint operational proposals of the International Labor Organization (ILO, Genava, Switzerland) and the World Health Organization (WHO, Genava, Switzerland), an occupational stress questionnaire was developed and then validated in Spanish: the ILO-WHO stress scale [16].

However, with the exception of the validation study of the original instrument, these psychometric characteristics have been mostly investigated to date with samples of limited dimensionality (fewer than 1000 subjects, and mostly fewer than 500), using homogeneous samples from individual working sectors, using entirely exploratory analysis procedures, and exclusively in Latin America [36,37,38,39,40,41].

Thus, the aim of the present study was to analyze the evidence of the psychometric properties of the ILO-WHO stress scale and its validity in diagnosing work stress. For this reason, a study was designed using a large heterogeneous sample of workers from the Canary Islands. The testing of this international tool will allow the better implementation of stress assessment and diagnosis policies, and of the subsequent stress management and intervention phases.

## 3. Materials and Methods

### 3.1. Sample

The sample consisted of 1533 Canary Islands workers. Once the single-category responses were applied, 18 subjects were removed from the sample, leaving a definitive sample of 1515, aged between 18 and 76 years (M = 38.45; SD = 12.16), which was well balanced in terms of gender (men, n = 704, 46.5%; women, n = 811, 53.5%) and in years of work service (between 1 and 57 (M = 9.56; SD = 9745)). Nine categories of workers were determined according to their job tasks (health, police/military; teaching; hospitality; politics; services; self-employed; journalists; civil service) (Table 1).

### 3.2. Instrument

The evaluation of the psychometric properties of the ILO-WHO stress scale was carried out through a cross-sectional study of a large sample of workers.

The work stress scale is a quantitative support instrument provided by the International Labor Organization and the World Health Organization to detect work stress [38]. This scale has 25 items, which are related to the 7 segments of labor activity as reported by Ivancevich and Mattesson [16]: organizational climate, organizational structure, leader influence, lack of cohesion, territory, technology and group support. The scale is designed according to the Likert model, which has seven response options for each item, with options ranging from 0 (never) to 6 (always). This scale was published by Ivancevich and Matteson [16] to measure work-related stressors at three levels: organizational, group and individual. Originally called the “Stress Diagnostic Survey”, it was then modified to analyze only the two environmental dimensions (group and organizational stressors), removing the individual-level scale dimension (which consisted of 30 items). It was validated on a sample of 38,072 workers from all sectors and its usefulness lies in its ability to predict sources of psychosocial risks [42].

In an introductory section of the questionnaire, participants were asked to provide information on socio-demographic characteristics (e.g., gender, age, etc.) and job-related variables, such as role, position, and seniority.

### 3.3. Procedure

Respondents were mainly invited by email and the ILO-WHO scale was administered using the Google Form tool; subjects had to authorize the use of the data for research purposes and return the questionnaires. Workers were provided with a description of the study (e.g., methods, institutional affiliations of the PI) and were informed of their right to refuse to participate in the study or withdraw consent to participate at any time during the study without consequence. Participants then confirmed that they properly understood the instructions, gave their consent, and moved on to completing the questionnaire. The present study was conducted in accordance with the guidelines defined by the Declaration of Helsinki. The average time taken to complete the questionnaire was 9.39 min.

The overall sampling method was useful in that it was aimed at diversity in job sectors and profiles, with the intention of enhancing generalizability; the cross-national sampling strategy made use of a snowballing technique and workers were recruited by students taking the Labor Relations degree in the Faculty of Law of the University of La Laguna. Before administering the questionnaire, participants were informed that participation was anonymous and voluntary. Data were collected between September 2019 and June 2020. All participants provided information on their, age, gender, current position, academic qualifications and length of service before completing questionnaires.

The study was conducted in accordance with the Declaration of Helsinki and approved by the Institutional Review Board (or Ethics Committee) of the University of e-Campus (protocol code 03/2020, date of approval 28 December 2020).

### 3.4. Data Analysis

For statistical analysis, data went through an extensive and robust process of properties testing, with a combination of the techniques of exploratory factor analysis (EFA) and confirmatory factor analysis (CFA), with the aim of finding strong evidence of validation in the construction stage, and also its stability. EFA is a multivariate statistical method that attempts to identify the smallest number of hypothetical constructs (also known as factors, dimensions, latent variables, synthetic variables, or internal attributes) that can parsimoniously explain covariation [43]. EFA requires the fulfillment of several stages, including data inspection techniques, the factor analysis method, the identification of the underlying structure and rotation technique, and the factor quality indexes.

To test the dimensionality a robust parallel analysis was performed. This is considered one of the most effective and accurate techniques for testing the number of factors/dimensionalities and uses optimal implementation with a minimum rank factor analysis that minimizes the common variance of the residuals [44]. The adequacy of the polychoric correlation matrix was assessed using Bartlett’s statistical test and the Kaiser–Meyer–Olkin (KMO) test. In order to apply the CFA, it is necessary that the sample is different from the one used for the EFA. The Solomon method [45] was therefore employed to split the total sample into two equivalent subsamples, therefore the total sample of 1515 was split into two groups: a first subsample with 758 individuals, and a second with 757. To assess how equivalent the two subsamples were, the ratio communality index (RCI) was used and applied as follows: the closer its value to 1, the more equivalent the subsamples are. The robustness of the test was determined by associating a bootstrap with a sample extrapolation to 500 units. The factors were extracted using the RULS (robust unweighted least-squares) technique, which reduces the residuals of the matrices.

For the CFA adjustment indices, factor loads greater than 0.50 and the following minimum indices for adequacy were considered, taking into account the numbers of participants (1515) and variables (25): NNFI (non-normed fit index) >0.95; CFI (comparative fit index) >0.95; BIC (Schwarz’s Bayesian information criterion) 90% confidence interval; GFI (goodness-of-fit index) >0.95; AGFI (adjusted goodness-of-fit index) >0.95; RMSEA (root mean square error of approximation) <0.05, and RMSR (root mean square of residuals) <0.05. It should be noted that the expected mean value of the RMSR should not be higher than Kelly’s criterion if it is to be considered an adequate fit.

As a complementary analysis to test the number of factors, the following techniques of unidimensionality/multidimensionality were applied: UNICO (unidimensional congruence) >0.95; I-Unico (item unidimensional congruence) >0.95; ECV (explained common variance) >0.80; I-ECV (item explained common variance) >0.85; MIREAL (mean of item-residual absolute loadings) <0.30, and I-REAL (item residual absolute loadings) >0.30. These techniques were applied to the instrument and to the items. For the items, they were used to assess whether the item would adhere in a unidimensional or multidimensional manner; that is, if there was a possibility that the item would load significantly in more than one dimension.

The replicability of the construct was assessed by the generalized G-H Index [46] with an index value greater than 0.80 suggesting a well-defined latent variable. For the quality and effectiveness of the factor estimation, the factor determinacy index (FDI) was used, pointing to an adequate estimate with values greater than 0.90.

Other indicators measuring quality and effectiveness of factor score estimates also provided evidence on the one-dimensionality of the workplace stressors constructs measured in the questionnaire; the sensitive ratio (SR > 2) can be interpreted as the number of different factor levels that can be differentiated on the basis of the factor score estimates. The expected a posteriori (EAP > 0.80) score indicates marginal reliability. The expected percentage of true differences (EPTD > 90%) represents the estimated percentage of differences between the observed factor score estimates that are in the same direction and the corresponding true differences [47].

The descriptive statistics dataset was analyzed through the IBM SPSS v.22 (New York, NY, USA) statistical package and to analyze structure dimension a specialized factor analysis was applied called FACTOR v.12.01.02 [48,49,50,51].

## 4. Results

### 4.1. Preliminary Analysis

No outliers were identified by SPSS software analysis of the whole sample. To analyze asymmetry and kurtosis of the data, Mardia’s [52] multivariate descriptive was applied. It revealed a coefficient of kurtosis = 905.286 (critical ratio = 121.977; df = 2925; *p* < 0.05) with values from −1.384 to −0.982 and a skewness = 38.040 (critical ratio = 9605.049; df = 2925; *p* < 0.05) with values from −0.041 to 0.769. Given this non-normality, as well as the ordinal nature of the ILO-WHO items, a polychoric correlation matrix was deemed to be an appropriate input for EFA. The results of Bartlett’s test of sphericity indicated the adequacy of the polychoric correlation matrix which showed excellent values.

The qualification of the sample was analyzed in terms of the level of organizational stress suffered, as determined by the quartile distribution of the participants’ scores. Four levels of stress were categorized: the first level as low, the second level as medium, the third level as high and the fourth as very high (Table 2).

### 4.2. Exploratory Factor Analysis

An exploratory factor analysis (EFA) of the ILO-WHO 25-item scale using FACTOR software was carried out; results of the univariate descriptive analysis are displayed in Table 3. The scale revealed an optimal coefficient of internal consistency (Cronbach’s alpha = 0.961).

A preliminary analysis with no indication of number of factors to be extracted was carried out and the program advised us to treat the scale as a one-dimensional scale. Therefore, we instructed the program to treat the scale as one-dimensional and the whole analysis was carried out again with one factor, as previously advised by the cited software.

FACTOR allows a closeness to unidimensionality option to deal with the results of the assessment of the scale more precisely [47]. The one-factor solution was saliently loaded by the total set of 25 items.

Values of relative difficulty index (RDI) items should range between 0.40 and 0.60, and those of measures of sampling adequacy (MSA)—a useful index for debugging inappropriate items before a factor analysis solution—should all be above 0.50, suggesting that each item measures the same domain as the remaining items in the pool, and so should not be removed [48]. Item 22 would thus be the only item occupying the first quartile, while items 15, 2, 16, 14, 1, 13, 9, 8, 20, 17, 5, 12, 21, and 19 would occupy the second quartile and items 23, 7, 11, 3, 6, 24, 25, 10, 4, and 18 would be part of the third quartile. Overall, the lower the level in the quartile, the lower the level of difficulty of the item, as indicated in Table 4.

Table 5 shows the values of the factorial loads and commonality. The factorial loads ranged between 0.44 and 0.77 in the sample, which indicates satisfactory and adequate levels. In addition, commonalities values ranged between 0.26 and 0.61, with the exception of item 22 (0.199). All the rest show adequate values in the unrotated loading matrix, due to one-dimensionality structure.

For a better reading of the data, only the first 10 variables are shown in Table 6 and Table 7. Table 6 shows the values of explained variance based on eigenvalues. A first variable with an eigenvalue of 12.290 stands out from the other variables. Adding a second variable adds only 5% of the explained variance, therefore it is recommended to take only one variable in the structure.

The results of the parallel analysis (PA) based on minimum rank factor analysis is shown in Table 7. According to Timmerman and Lorenzo-Seva [44]: one single factor accounted for 53.47% of the total variance, which is an excellent percentage for explanation purposes given its unidimensional nature. Given these results, the one-factor solution can be considered as the most adequate structural representation of the ILO-WHO scale in the present sample and as robust across alternative extraction methods.

### 4.3. Confirmatory Factor Analysis

**Figure 1:**
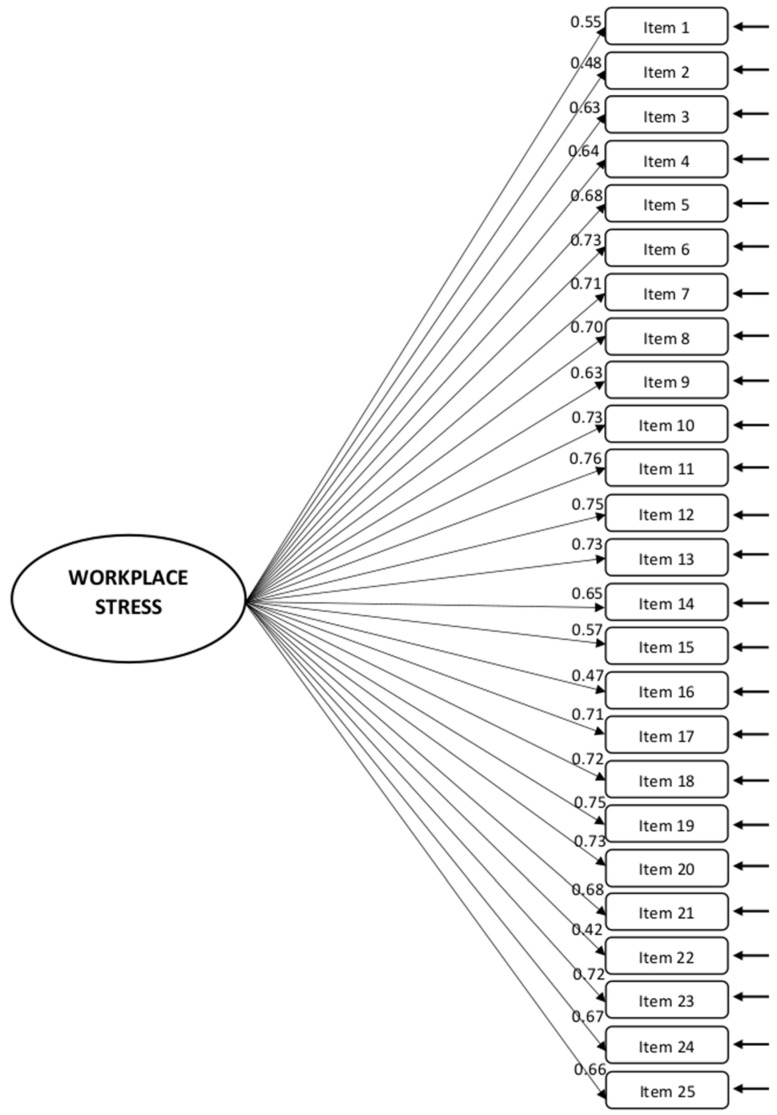
Final Factor Model for the 25-item ILO-WHO Workplace Stress Scale.

To verify the factor structure identified through EFA, a confirmatory factor analysis was performed on the holdout Solomon sample (N = 755). The 25 items were modeled as reflective indicators of the single factor extracted. Robust goodness-of-fit statistics were assessed using different fit indices, including chi-square, RMSEA, NCP, NNFI, CFI, BIC, GFI, and AGFI [53]. Thus, the CFA result cross-validated the single factor structure devised in the EFA (Table 8 and Figure 1).

## 5. Discussion

Organizational stress is currently a growing phenomenon in the workplace. In Spain, and more specifically in the Canary Islands, the changing living conditions of the population, a loss of social value, economic difficulties, and the arrival of immigrants on its coasts have had a permanent effect on working life. The number of people who have lost their jobs is very high and this has conditioned the perception that workers have of their work, increasing their perceived levels of stress [10].

The aim of this study was to illustrate with empirical evidence the factor structure of the ILO-WHO stress scale, it also sought to match the factor structure scale proposed by Ivancevich and Matteson [16] for organizational stress, initially composed of seven factors with a total of 25 items. The first factor is named “Organizational climate” and consists of four items (1, 10, 11, and 20). The second factor is “Organizational structure”, and comprises four items (2, 12, 16, and 24). The third factor is called “Organizational territory” and contains four items (3, 15, and 22). The fourth factor is called “Technology”, and consists of three items (4, 14, and 25). The fifth factor is “Leader influence” and is composed of four items (5, 6, 13, and 17). The sixth factor is called “Lack of Cohesiveness”, and consists of four items (7, 9, 18, and 21). The seventh factor is named “Group Support” and contains three items (8, 19 and 23).

With this distribution of factors, we set out to verify the factor structure that would explain the greatest possible variance. The first step was to analyze the internal structure of the scale proposed by Ivancevich and Matteson [16] by initially using the EFA and subsequently ratifying the structure—with a different sample—through the CFA.

Based on this validated factor model, the analysis proceeded to examine the content of the 25 ILO-WHO items, which allowed us to carry out a precise analysis of the factorial structure scale, which resulted in our assigning the label of ‘Organizational and group stress’ to the single factor found. Using the evidence-based EFA method, the study found that the 25-item scale suggests a one-factor model to explain the total variance. Subsequent studies found two factors in some instances and three factors were identified in others.

Except for the validation study, no research has yet been able to confirm the multi-factorial nature of the tool or find a correspondence between extracted factors and the organizational dimensions of stress mentioned by the authors. It is certainly necessary to bear in mind the fact that the studies in question used the instrument in Spanish-speaking contexts, mostly Latin American (Peru, Mexico, Ecuador, etc.), with samples that were often homogeneous and limited in size (sometimes fewer than 250 subjects), and with statistical analysis procedures that were often entirely exploratory [36,37,38,39].

Overall, however, the previous research has shown a transversal two-factor structure, not always satisfactory for the variance explained (systematically lower than 45%), but with internal validity values of the measure at 25 items always higher than the value 0.91, and evidence validity for both construct and content.

The results of the present study demonstrated that scales for organizational and group stress showed unidimensional characteristics, satisfactory factor loadings and very good levels of reliability, evidence which points to an instrument with a consistent and reliable internal structure for measuring stress variables, and also provides the new insight that stress is perceived as a single dimension, regardless of the analysis of factor content.

As noted above, in considering the four stress ranges, the results of this study show a low-stress level for 25% of the sample (N =377); medium-stress level for 35.7% (N = 539); a high-stress level for 30.8% (465) and finally an extreme-stress level reported for just 8.5% (129). Bearing in mind the internal consistency of the scale, which shows an ordinal alpha α = 0.961, we can state without any doubt that the scale has very good reliability. Turning to the explained variance, we found that a one single factor accounted for 53.47% of the total variance, which represents a very good figure for a unidimensional factor. As is mentioned above, the value of the expected mean value of the RMSR should not be higher than Kelly’s criterion. The value of RMSR is 0.0537 and the expected mean value of the RMSR according to Kelly’s criterion is 0.0257; therefore, we can consider this an adequate one-dimension model fit.

It is important to bear in mind the importance of the confirmatory factor analysis results. Our data show excellent and robust results that allow us to affirm that we are here dealing with a unidimensional factor structure.

Among the strengths of this study, it should be noted that the fact that we used a large sample for the EFA and a different sample for the EFC makes our results, using specialized factor analysis software, highly useful for the future. These studies should be repeated with other samples of subjects, given that all the studies that have been carried out to date have involved Hispanic populations, both in Latin America and in Spain. Therefore, to understand if the factorial structure is maintained, a different sample would have to be used. Despite being a construct that has been studied for some time, the construct of work stress has been analyzed by different theoretical and methodological approaches, giving rise to a sort of multiplication of formulations that show overall the substantial multidimensional nature of both work stressors and stress as an outcome; this premise also allows us to understand the difficulty of achieving a certain level of homogeneity in construct measurement.

As well as considering these matters, and in order to adequately assess the results of our present research, it is necessary to mention the following limitations of the study: firstly, the nature of the sampling process, while convenient, certainly limits the generalization of the results. In addition, it would have been very useful to be able to evaluate the convergent validity results with similar constructs or measures, so future research might also use other alternative stress measures and constructs in parallel. Further verification of the psychometric characteristics might also be evaluated with other statistical approaches such as partial least squares (PLS) [54] Another limitation of this study stems from the heterogeneity of the sample with respect to the work sectors to which individuals belonged, which did not allow for an adequate comparison between groups. We therefore envisage future studies with independent sub-samples defined by working sector or role.

Workplace stress measures may vary across contexts, organizations and circumstances of assessment, and stress might be measured once and expected to remain constant over a considerable period, as part of a preventive measures recommended to ameliorate well-being at work and productivity. In practice, the planning of workplace stress assessment could have a significant impact on the individual and relational behavior of employees, and could help in the prevention of counterproductive behaviors, turnover, absenteeism and conflicts [55].

## 6. Conclusions

The 25-item ILO-WHO scale is a reliable and valid scale for measuring organizational and group stress amongst workers from the Canary Islands.

Our results underline the value of implementing stress measures and offer new perspectives for intervention that could be planned from a preventive perspective to promote organizational well-being that could be increased through specific training.

This is particularly important for workers from the Canary Islands, where changing economic circumstances and living conditions have increased perceived levels of stress. A focus on stress reduction measures could be adapted to the specific needs of Canary Islands workers or to promote health and well-being in other, different contexts. However, as with any correlational design research, we must be cautious when interpreting results, and more tests should be carried out amongst other groups, and in other countries, in order to better validate the scale.

## Figures and Tables

**Table 1:** Demographics variables of the sample.

Age Group		Frequency	
Interval	18–30	513	33.9%
	31–40	351	23.2%
	41–50	355	23.4%
	51–75	296	19.5%
	Total	1515	100.0%
Specialty		Frequency	
Health worker		179	11.8%
Police/military		59	3.9%
Teaching		128	8.4%
Hospitality		219	14.5%
Politician		51	3.4%
Services		624	41.2%
Self-employed		126	8.3%
Journalist		62	4.1%
Civil servant		67	4.4%
Total		1515	100.0%

**Table 2:** Distribution of grouped organizational stress scores in quartiles.

	Freq.	%	Cumulative %
Low stress <48	379	25.0	25.0
Medium stress 48–80	539	35.8	60.7
High stress 81–114	465	30.7	91.5
Ext. high stress 115–147	129	8.5	100.0
Total	1515	100.0	

**Table 3:** Descriptive statistics and pattern coefficients.

Items	Mean	Variance	Skewness	Kurtosis
1	2.701	2.755	0.158	−0.793
2	2.136	2.684	0.472	−0.622
3	3.136	2.718	−0.041	−0.828
4	3.368	2.831	−0.174	−0.775
5	2.791	3.996	0.109	−1.216
6	3.212	4.722	−0.120	−1.384
7	2.881	3.353	0.071	−1.024
8	2.743	3.055	0.189	−0.896
9	2.728	3.452	0.153	−1.064
10	3.308	3.285	−0.139	−1.020
11	3.128	3.390	−0.089	−1.034
12	2.814	3.144	0.073	−0.982
13	2.723	3.809	0.161	−1.122
14	2.686	3.378	0.130	−1.039
15	2.079	3.271	0.557	−0.750
16	2.319	3.352	0.401	−0.892
17	2.778	4.059	0.154	−1.225
18	3.487	3.219	−0.155	−1.059
19	2.830	3.460	0.126	−1.064
20	2.772	3.736	0.115	−1.120
21	2.814	3.595	0.142	−1.072
22	1.670	2.617	0.769	−0.266
23	2.865	3.214	0.062	−1.016
24	3.262	3.695	−0.155	−1.115
25	3.284	3.128	−0.153	−0.892

**Table 4:** Item location and item adequacy.

Items	QIM	RDI	Normed MSA
22	1	0.27811	0.92566
15	2	0.34675	0.95515
2	2	0.35622	0.94360
16	2	0.38625	0.95879
14	2	0.44730	0.96618
1	2	0.44950	0.96448
13	2	0.45325	0.97749
9	2	0.45413	0.97021
8	2	0.45710	0.96614
20	2	0.46128	0.97369
17	2	0.46326	0.97292
5	2	0.46491	0.94550
12	2	0.46876	0.97716
21	2	0.46909	0.97524
19	2	0.47129	0.97884
23	3	0.47734	0.97013
7	3	0.47921	0.97353
11	3	0.52090	0.96771
3	3	0.53058	0.95701
6	3	0.53476	0.94803
24	3	0.54345	0.96978
25	3	0.54675	0.96597
10	3	0.55116	0.96498
4	3	0.56062	0.95170
18	3	0.58042	0.97675

**Table 5:** Values of unrotated loading matrix.

Items	Factor Loadings	Commonality
1	0.575	0.330
2	0.514	0.264
3	0.650	0.423
4	0.655	0.429
5	0.706	0.498
6	0.768	0.590
7	0.726	0.527
8	0.721	0.520
9	0.648	0.420
10	0.738	0.545
11	0.775	0.601
12	0.764	0.584
13	0.763	0.582
14	0.677	0.458
15	0.605	0.366
16	0.483	0.233
17	0.737	0.543
18	0.728	0.530
19	0.772	0.596
20	0.750	0.562
21	0.708	0.501
22	0.446	0.199
23	0.742	0.551
24	0.688	0.473
25	0.680	0.462

**Table 6:** Explained variance based on eigenvalues.

Variable	Eigenvalue	Proportion of Variance
1	12.29098	0.49164
2	1.45064	0.05803
3	1.09845	0.04394
4	0.86477	0.03459
5	0.78951	0.03158
6	0.76851	0.03074
7	0.68230	0.02729
8	0.62669	0.02507
9	0.57489	0.02300
10	0.55948	0.02238

**Table 7:** Parallel Analysis based on minimum rank factor analysis.

Variable	Real-Data % of Variance	Mean of Random % of Variance
1	53.4740	7.9800
2	6.1649	7.6026
3	4.5869	7.2551
4	3.4223	6.9113
5	3.2188	6.5699
6	3.1430	6.2230
7	2.6936	5.8948
8	2.4661	5.5880
9	2.2449	5.2606
10	2.1591	4.9536

**Table 8:** Synthesis of the model for the ILO-WHO instrument.

Adequacy of Correlation Matrix <0.000001		
Determinant of the matrix (Bartlett)	17,325.0	df = 300
KMO (Kaiser–Meyer–Olkin)	0.9656	very good
		
Exploratory factor analysis (first subsample)	N = 758	
Explained variance based on eigenvalues	0.49164	
Real-data percentage of explained variance	53.47%	
Robust mean and variance adjusted chi-square	1585.266	df = 275
Goodness-of-fit index chi-square	18.29.591	df = 255; sig 0.000
		
Confirmatory factor analysis (second subsample)	n = 757	
Non-normed fit index (NNFI)	0.986	
Comparative fit index (CFI)	0.987	
Goodness-of-fit index (GFI)	0.989	
Adjusted goodness-of-fit index (AGFI)	0.988	
Schwarz’s Bayesian information criterion (BIC)	1951.424	
Root mean square error of approximation (RMSEA)	0.056	
		
Distribution of residuals		
Number of residuals	300	
Root mean square of residuals (RMSR)	0.0537	
Weighted root mean square of residuals (WRMSR)	0.0580	
Expected mean value of the RMSR for an acceptable model (Kelly’s criterion)	0.0257	
		
Reliability		
Standardized Cronbach’s alpha	0.961	
Construct replicability–generalized H (GH) (H-Latent)	0.96	
		
Unidimensionality (overall assessment)		
Unidimensional congruence (UNICO)	0.968	
Explained common variance (ECV)	0.915	
Mean of item residual absolute loading (MIREAL)	0.165	
		
Quality and effectiveness of factor score estimates		
Factor determinacy index (FDI)	0.98	
EAP marginal reliability	0.96	
Sensitivity ratio (SR)	4.923	
Expected percentage of true differences (EPTD)	96.1%	

## Data Availability

Not applicable.
